# Clonal dynamics studied in cultured induced pluripotent stem cells reveal major growth imbalances within a few weeks

**DOI:** 10.1186/s13287-018-0893-2

**Published:** 2018-06-18

**Authors:** David Brenière-Letuffe, Aya Domke-Shibamiya, Arne Hansen, Thomas Eschenhagen, Boris Fehse, Kristoffer Riecken, Justus Stenzig

**Affiliations:** 1 Department of Experimental Pharmacology and Toxicology, University Medical Center Hamburg–Eppendorf, Martinistrasse 52, 20246 Hamburg, Germany; 2 Core Facility Stem Cells, University Medical Center Hamburg–Eppendorf, Martinistrasse 52, 20246 Hamburg, Germany; 3 DZHK (German Centre for Cardiovascular Research), partner site Hamburg/Kiel/Lübeck, Hamburg, Germany; 4 Research Department Cell and Gene Therapy, Department of Stem Cell Transplantation, University Medical Center Hamburg–Eppendorf, Martinistrasse 52, 20246 Hamburg, Germany

**Keywords:** Induced pluripotent stem cells, Stem cell culture, RGB marking, Clonal diversity, Induced pluripotent stem cell quality

## Abstract

**Background:**

Human induced pluripotent stem (iPS) cells have revolutionised research and spark hopes for future tissue replacement therapies. To obtain high cell numbers, iPS cells can be expanded indefinitely. However, as long-term expansion can compromise cell integrity and quality, we set out to assess potential reduction of clonal diversity by inherent growth imbalances.

**Methods:**

Using red, green, blue marking as a lentiviral multi-colour clonal cell tracking technology, we marked three different iPS cell lines as well as three other cell lines, assigning a unique fluorescent colour to each cell at one point in culture. Subsequently, we followed the sub-clonal distribution over time by flow cytometry and fluorescence microscopy analysis in regular intervals.

**Results:**

In three human iPS cell lines as well as primary human fibroblasts and two widely used human cell lines as controls (K562 and HEK 293 T), we observed a marked reduction in sub-clonal diversity over time of culture (weeks). After 38 passages, all iPS cultures consisted of less than 10 residual clones. Karyotype and function, the latter assessed by cardiomyocyte differentiation and tissue engineering, did not reveal obvious differences.

**Conclusions:**

Our results argue for a quick selection of sub-clones with a growth advantage and flag a normally invisible and potentially undesired behaviour of cultured iPS cells, especially when using long-term cultured iPS cells for experiments or even in-vivo applications.

**Electronic supplementary material:**

The online version of this article (10.1186/s13287-018-0893-2) contains supplementary material, which is available to authorized users.

## Background

Since the isolation of the first pluripotent stem cells by Martin and by Evans and Kaufman in 1981 [1, 2], the scientific community has been interested in the cells’ unique properties and potential. The discovery of human induced pluripotent stem cells (hiPSCs) by Takahashi and Yamanaka in 2006 [3] added additional momentum to an already extremely fast expanding field. However, obtaining and maintaining stem cells of sufficient quality is still a challenge. Numerous studies have shown that the genetic and epigenetic quality of hiPSCs does not meet medical standards and is even rapidly decreasing over time during routine cell culture [4–8]. These studies find that genetic mutations, duplications and deletions are common in both iPS and ES cell culture. In parallel, another field of research focuses on cell-to-cell variability in a single culture dish. It was first discovered in *Escherichia coli* that the expression of two different fluorophores under control of identical promoters was subject to variation (noise) over time. This experiment was the first to demonstrate stochastic behaviour of gene expression in a theoretically uniform *E. coli* culture [9]. These experiments were then reproduced in eukaryotes and demonstrated that gene expression was more variable than previously anticipated and regulated in a burst-like manner [10, 11]. Similarly, an iPSC culture is thought to be of monoclonal origin, consisting of largely identical cells. However, during cell culture slight stochastic changes within individual cells might confer a competitive advantage to one cell compared to the rest of the culture. These dynamics are potentially problematic during long-term cell culture in which cell-to-cell competition further amplifies sub-clones with additional stochastic genetic or epigenetic variations acquired during cell culture. The need to maintain high stem cell quality in regenerative medicine or tissue engineering raises the question of whether and how cell quality changes during the culture process. Not many tools are readily available to monitor and analyse the dynamics of this process. We therefore employed RGB marking with Lentiviral Gene Ontology (LeGO) vectors [12, 13] as an informative and easy-to-use method to visually analyse the clonal composition in cell culture. RGB marking introduces an artificial polyclonality by labelling each and every cell with a unique inheritable colour which is then transmitted to the cell’s progeny, making it an excellent tracking tool for cell sub-clones. Fluorophores expressed by the LeGO vectors are stochastically mixed within individual cells, which is due to a random number of vector integrations per cell and due to a random brightness per colour as a consequence of integration site variation of the lentiviral vectors. The latter can lead to great differences in expression of the fluorescent protein depending on chromatin accessibility and structure at the integration site. Lentiviral vectors stably integrate into the cell’s genome, and thus a single cell and its daughter cells will retain their individual colour during a potentially unlimited number of cell divisions, allowing for clonal tracking by colour. The fluorophores themselves will also underlie stochastic, cell cycle-dependent and culture condition-dependent changes in expression level over time. However, the simplicity of regulation of the expression cassettes without involvement in complex regulatory networks, in combination with the strength of the promoter and the long half-life of the fluorophores (levelling the protein amount over several days), ensures similar expression in cells derived from the same clone, allowing for the discrimination of individual clones in both fluorescence microscopy and flow cytometry [13].

We aimed at visualising the dynamics of this otherwise unnoticed phenomenon of clone number reduction and clonal overgrowth in hiPSC culture using this simple yet effective tool. The suitability of RGB marking for this purpose has been confirmed in mesenchymal stromal cells by genetic barcoding [14]. In our hands, clonal overgrowth in iPS cell culture could indeed be reproducibly demonstrated by both fluorescence microscopy and flow cytometry. This study highlights the need for a careful control of iPS cell culture quality in view of the potential outgrowth of a clonal sub-type in the culture dish. This observation is of special importance for those aiming to use iPSCs as a tool for drug development, regenerative medicine, cell therapy or tissue engineering as, for example, cells with a pre-existing growth advantage might be especially prone to teratoma formation and de-differentiation.

## Methods

### iPS cell culture

Three iPSC lines were used: C25-young, C25-old and BJ. The C25 cell line was kindly provided by Alessandra Moretti (Munich, Germany). The C25-young cell line was passaged 48 times before the experiment, while C25-old was passaged 112 times. The BJ cell line was provided by the Stem Cell Core facility (UKE, Hamburg, Germany) and was passaged 40 times before the experiment. Cells were cultured in FTDA medium (DMEM/F12 supplemented with l-glutamine, transferrin selenium mix, human serum albumin solution, lipid mix, insulin, dorsomorphine, activin A, TGF-β1 and FGF [15]), in six-well plates with 2.5 ml of medium and daily medium change. Passaging was performed at a 1:6 ratio whenever cell confluency reached 90%. However, this ratio was adjusted to as low as 1:3 in order to synchronise the different cell lines if one cell line was proliferating slower.

Passaging was performed with 0.5 mM EDTA. After 10 min of incubation, cell detachment was stopped by adding FTDA medium in excess. The detached cells were collected, centrifuged and resuspended in FTDA medium to be cultured further at 37 °C, 90% humidity and 5% CO_2_.

### RGB marking of iPS cells

RGB marking of cells for clonal tracking was performed as described previously [13, 16]. For the labelling of iPS cells, 150,000 cells per well of a 12-well cell culture plate were plated in 1 ml medium. RGB marking was carried out using concentrated stocks of three VSV-G pseudotyped LeGO vectors, each expressing a fluorescent protein of one of the basic colours under control of an EF1α promoter, linked to a puromycin resistance by a 2A sequence: LeGO-EF1a-B2-Puro^+^ (expressing mTagBFP, blue), LeGO-EF1a-V2-Puro^+^ (expressing Venus, green) and LeGO-EF1a-C2-Puro^+^ (expressing mCherry, red) [12, 17]. Functional titres were in the range of 3.5 × 10^8^–6.2 × 10^8^/ml, titrated on 293 T cells. A multiplicity of infection (MOI, virus genomes per cell) of 25, relative to the number of 293 T cells, was needed to obtain transduction rates of 50–70% per colour within the transduced proportion of the cells. Note that the apparently high MOI relative to 293 T cells reflects the lower susceptibility of iPS cells towards transduction. An absolute MOI of about 3 (MOI 1 per colour) is needed for optimal colour distribution within the RGB-marked cells, with MOI 1 leading to about 63% positive cells following the Poisson distribution [18]. Selection of transduced cells was done by addition of 1 μg/ml puromycin.

### Differentiation of iPS cells to cardiomyocytes and tissue engineering

The differentiation of RGB-marked iPSCs to cardiomyocytes and the generation of engineered heart tissue (EHT) were performed as described previously [19]. In brief, differentiation was achieved by spinner flask-based embryoid body (EB) formation, followed by mesoderm induction with BMP4, activin A and bFGF, and subsequent Wnt-signalling inhibition for cardiac differentiation. EBs were dissociated with collagenase II to generate a single-cell solution for EHT casting. Each EHT was cast in a 24-well 3D cardiomyocyte culture format, in which the cells were embedded in a strip of fibrin matrix attached to flexible silicone mounting posts at both ends. EHTs would then beat spontaneously and produce force, regularly deflecting the posts after 10–14 days. Cells were counted manually. For each EHT, 500,000 cells were mixed with fibrinogen and thrombin was added to initiate polymerisation in a volume of 100 μL per tissue construct. The mix was then quickly transferred into a casting mould in a 24-well cell culture dish with silicone racks placed on top. The cells were then left for 90 min at 37 °C to ensure full fibrin polymerisation, before being transferred to a new 24-well plate containing fresh DMEM-based medium. EHTs were kept for a maturation period of 2 weeks with medium change every other day before contractility was analysed using a custom-designed incubator equipped with a movable camera setup and pattern recognition software.

### RGB marking of primary human dermal fibroblasts

For the labelling of primary human dermal fibroblasts (HDFa; Thermo Fisher), 100,000 cells per well of a 12-well cell culture plate were plated in 1 ml medium (DMEM with glutamine and 4.5 g/L glucose supplemented with 100 U/ml penicillin, 100 μg/ml streptomycin and 10% horse serum). RGB marking was carried out using the same concentrated stocks of LeGO vectors that were used for transduction of iPS cells. An MOI of 1, 2, 3 or 10 (relative to the number of 293 T cells) was used to obtain average transduction rates of 33% (MOI 1) to 45–50% (MOI 2, 3 or 10) per colour. Selection of transduced cells was achieved by addition of 1 μg/ml puromycin.

### RGB marking of 293 T and K562 cell culture

In a 24-well plate, 50,000 293 T cells (ATCC CRL-11268) or K562 cells (ATCC CCL-243) per well in 500 μl medium (DMEM or RPMI respectively) were transduced with equimolar amounts of three lentiviral vectors at the same time at different MOI values per well. Each vector was designed to express a fluorescent protein in one of the three basic colours under control of the SFFV promoter: LeGO-B2 (expressing mTagBFP, blue), LeGO-V2 (expressing Venus, green) and LeGO-C2 (expressing mCherry, red). Cells were analysed by flow cytometry 7 days later and the well with the desired transduction rate of 50–70% per colour was selected for the experiments, MOI 4 in the case of 293 T cells and MOI 16 in case of K562 cells.

### Flow cytometry and fluorescence microscopy

An LSRFortessa flow cytometer (BD, Heidelberg, Germany) was used for flow cytometry, equipped with three lasers (405 nm, 488 nm, 561 nm) to optimally excite the three fluorescent proteins. The bandpass filters were 450/50 nm to measure mTagBFP, 530/30 nm for Venus and 610/20 nm for mCherry. An IX81 motorised fluorescence microscope (Olympus, Hamburg, Germany) was used to image the RGB-marked cells, equipped with a 4× objective and a ColorView II camera. Several pictures were taken and stitched (8 × 8 images) per well using the Image Composite Editor software (Microsoft, Redmond, WA, USA) to increase the recorded area per well.

## Results

### Flow cytometry demonstrates clonal overgrowth in iPS cell culture

In order to investigate clonal dynamics during routine iPSC culture by RGB marking, iPSCs were lentivirally transduced to express stochastic combinations of three fluorescent proteins in the basic colours red, green and blue [13]. Two iPSC lines were used: BJ reprogrammed from commercially available foreskin fibroblasts by Sendai virus-based iPS induction; and C25 reprogrammed from skin fibroblasts using the more classical lentiviral vector approach. To additionally investigate a possible difference in clonal dynamics depending on the age of the cell culture, the C25 cell line was labelled at two different passage numbers, passage 48 (C25-young) and passage 112 (C25-old). The BJ cell line was labelled at passage 40, similar to C25-young.

For all three iPS cell lines the transduction rate was adjusted to 60–70% per colour, to obtain an optimal colour distribution by RGB marking (Table 1). To obtain a pure labelled population, puromycin was used for a few passages to enrich the RGB-marked cells. Almost 100% of the remaining cells were labelled by an individual colour.

**Table 1:** Initial flow cytometry of 30,000 cells after RGB marking

	C25-old cells, passage 6		BJ cells, passage 6		C25-young cells, passage 8	
	Events	% total	Events	% total	Events	% total
mTagBFP	18,442	61.5	17,615	58.7	20,134	67.1
Venus	18,873	62.9	18,136	60.5	18,346	61.2
mCherry	18,848	62.8	17,143	57.2	20,282	67.6

*BFP* blue fluorescent protein

**Fig. 1:**
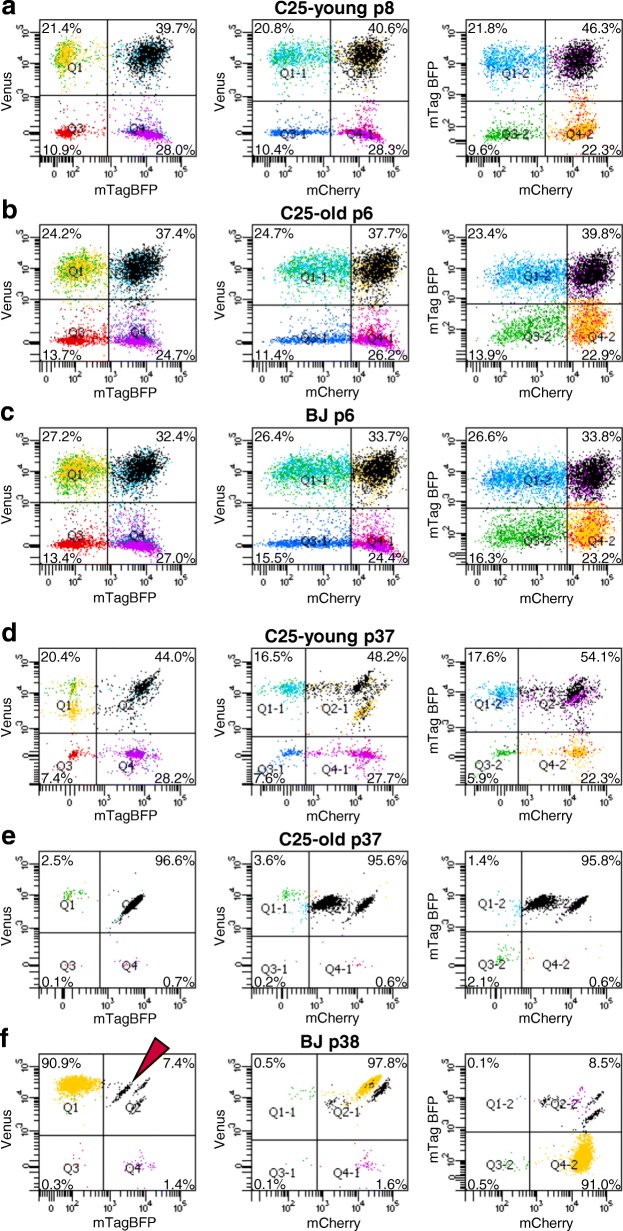
Flow cytometry of RGB-marked iPS cell lines at early passage and 37–38 passages after transduction. Left to right: green plotted against blue channel, green against red and blue against red. MOI 25 used for RGB marking. **a** C25-young cells at passage 8. Homogeneous distribution of dots within dot plots without any distinguishable clones indicates a polyclonal state. **b** C25-old cells at passage 6. A polyclonal state can be observed. **c** BJ cells at passage 6. A polyclonal state can be observed. **d** C25-young cells 37 passages after RGB marking. Oblique lines in top-right quadrant indicate presence of only a few clones. **e** C25-old cells 37 passages after RGB marking. Two clones have overtaken almost the entire culture, representing 95% total cells. **f** BJ cells 38 passages after RGB marking. Yellow clone represents 90% of cells, while rest mostly represented by four clones. Arrowhead points out one streak representing a single-coloured minor clone. BFP blue fluorescent protein, p passage, Q quadrant

Flow cytometry analysis at passage 6 or 8 after RGB marking showed a cloud-like distribution for every cell line, indicating a polyclonal state and random colour labelling at this stage. Because of the high number of clones present at this stage, it was not possible to distinguish individual cell clones in any of the three lines (Fig. 1a–c). Subsequently, the cell cultures were analysed by flow cytometry at regular intervals during routine cell culture. The change from a polyclonal to an oligoclonal population is marked by the gradual development of a characteristic streaky pattern in the top right quadrant of 2D flow cytometry plots. This pattern is caused by single clones becoming dominant in the culture, as we have shown previously by analysing a defined mixture of single-cell-derived clones [20]. The pattern can be explained by different fluorescence intensities (the brightness) among the cells of one clone due to technical and biological variation (length of each streak), while the relation of the two fluorophores (the colour) stays remarkably constant (width of the streak). Flow cytometry revealed that BJ cells started to become oligoclonal at passage 12 after RGB marking, C25-old cells at passage 16 and C25-young cells at passage 15. The number of colours, representing the number of clones in each cell line, continued to decrease until only very few clones were left at passages 37 and 38 (Fig. 1d–f). By the end of the culture period, all cell lines became oligoclonal or almost monoclonal with 90% of BJ cells derived from a single yellow clone, 95% of C25-old cells composed of only two different clones and C25-young cells still in an oligoclonal state in which clones could not be counted by our method.

Using flow cytometry analysis, we observed that all of the three cell lines were in an at least oligoclonal state around 15 passages after RGB marking. In both BJ and C25-old cell lines, only one or two clones were left after 37 passages. The clonal reduction in C25-young cells was less pronounced. Despite being simply a younger version of C25-old cells, this could be explained by the lower cell division rate of C25-young. Indeed, during the experiment, the splitting ratio of the C25-young cell line had to be lowered for technical reasons in order to keep all cell lines synchronously at the same passage number.

### Fluorescence microscopic images reveal clonal overgrowth in iPS cell culture

**Fig. 2:**
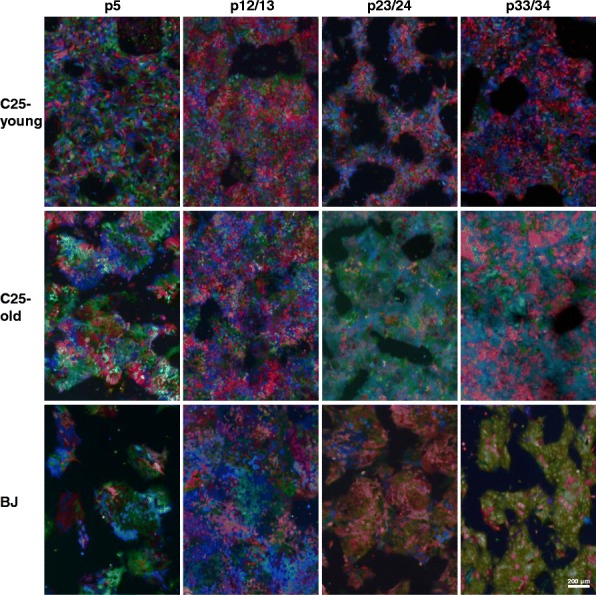
Fluorescence microscopy images of different cell lines at different passages. C25-young cells remain in an oligoclonal state until passage 23, which cannot be assessed in fluorescence microscopy. Note overgrowth of one or two colours in both C25-old and BJ cell lines. In C25-old cells, a mix of a grey/blue and a purple clone can be detected; while in BJ cells, a single clone of yellow/gold colour has almost overtaken the cell culture. Decrease in colour diversity detected from passage 23/24 onwards. C25-young cells stay in oligoclonal state until passage 23 which cannot be deduced from these microscopic images (compare Fig. 1). Scale bar indicates 200 nm. p passage

In parallel to flow cytometry, fluorescence microscopic images were taken at regular intervals. Although somewhat less obvious than in the flow cytometry experiments, the same conclusion could be reached based on the fluorescence microscopy images. While numerous different colours could be observed during the first passages, cell lines C25-old and BJ clearly exhibited overgrowth of individual colours from passage 23/24 onwards. By passage 33, C25-old cells displayed almost exclusively a grey/blue and a purple clone; while by passage 34, BJ cells consisted almost exclusively of one yellow/gold-coloured clone. C25-young cells, however, did not reveal any obvious colour difference from the first to the last passage (Fig. 2), which is in good accordance with the data gained by flow cytometry.

### Karyotyping

Karyotyping is part of a consensus test panel for cell culture to assess cell quality. We therefore FACS sorted the three different major clones obtained from C25-old (C25-old#1 and C25-old#2) and BJ cell lines at the end of the long-term culture experiment to assess whether the growth advantage of the dominant sub-clones can be explained by karyotype aberrations. The other sub-clones present in the culture were too sparse to be sorted by flow cytometry and C25-young cells were still in an oligoclonal state, which precluded sorting of a dominant sub-clone. The karyotypes of all respective clones remained physiological (Additional file 1: Figure S1).

### Functional integrity of RGB-marked iPS cells

To assess the functional integrity of RGB-marked iPS cells, they were also used in a complex experiment comprising directed differentiation and tissue engineering. An established multi-step protocol served to confirm the quality of the cells and to prove that the RGB-marked cells’ physiology and pluripotency were similar to their unmarked counterparts. The cells were differentiated to a cardiomyocyte-like phenotype following a published differentiation protocol and subsequently used for the generation of engineered heart tissue (EHT) [19, 21, 22]. The process led to a perfectly contractile EHT produced with the same protocol as for unmarked cells (Additional file 2: Video S1). The force and frequency of the contractions were similar to other unmarked EHTs of the same cell line. Thus, we did not observe functional differences between RGB-marked and unmarked cells, arguing for a negligible effect of the lentiviral transduction on cell integrity.

### Clonal overgrowth in non-iPS cell lines

**Fig. 3:**
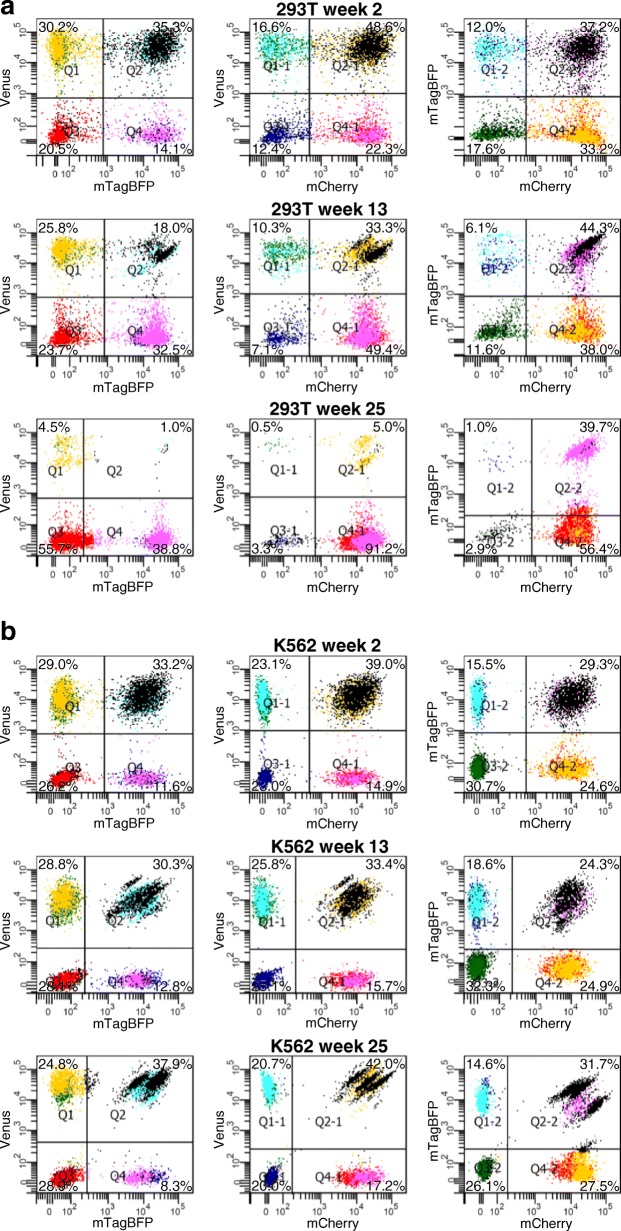
Flow cytometry of RGB-marked cell lines 293 T and K562 at different time points. Left to right in each panel: green plotted against blue channel, green against red and blue against red. MOI 4 and 16 used to transduce 293 T and K562, respectively. **a** Clonal dynamics of 293 T cells in one individual well of a six-well cell culture plate over 25 weeks. **b** Clonal dynamics of K562 cells in one individual well of a six-well cell culture plate over 25 weeks. Refer to Additional file 1: Figures S2 and S3 for full data of 24 time points within 6 months from other wells of six-well plate and from T75 flask. BFP blue fluorescent protein, Q quadrant

To compare the dynamics of clonal overgrowth in iPSCs to other cultured cells, we chose two human cell lines: the adherently growing 293 T cells; and the CML cell line K562 growing in suspension. These two cell lines were labelled by RGB marking of 50,000 cells, leading to about 44,000 individually labelled cells, each representing the starting point of an independently coloured clone (the total transduction rates were determined to be 92% and 85% respectively; Fig. 3, Additional file 1: Figures S2 and S3). In analogy to the experiments with iPSCs, the clonal behaviour of the cultures was followed by flow cytometry and fluorescence microscopy weekly for a period of half a year, yielding 25 data points per analysis method. To additionally analyse the influence of culture size, we cultured the cells both as a larger culture in T75 flasks and as about 10 times smaller cultures in three independent wells of a six-well plate per cell line. All four conditions were derived from the same RGB-marked culture. We hypothesised that a smaller culture size containing less individual clones would lead to oligoclonality in shorter time. The smaller the culture, the more likely a clone could get lost during the splitting and replating procedures of the routine culturing process. An extreme case would be splitting down to, for example, five cells, so that oligoclonality would already be reached by the first replating.

As expected, the highly polyclonal state in the beginning, represented by the homogeneous distribution of the data points in flow cytometric analyses, shifted to a state with the first dominant clones visible within 6–10 weeks. At the end of the observation period, after 20–25 weeks, the cultures became oligoclonal or even monoclonal (Fig. 3). The only exception was the large culture of 293 T cells (T75 flask) which did not show the appearance of dominant clones until the end of the observation period. In that case, the only observable difference was the decreasing proportion of blue or green cells, while red cells did not show a decrease.

### Clonal overgrowth in fibroblasts

**Fig. 4:**
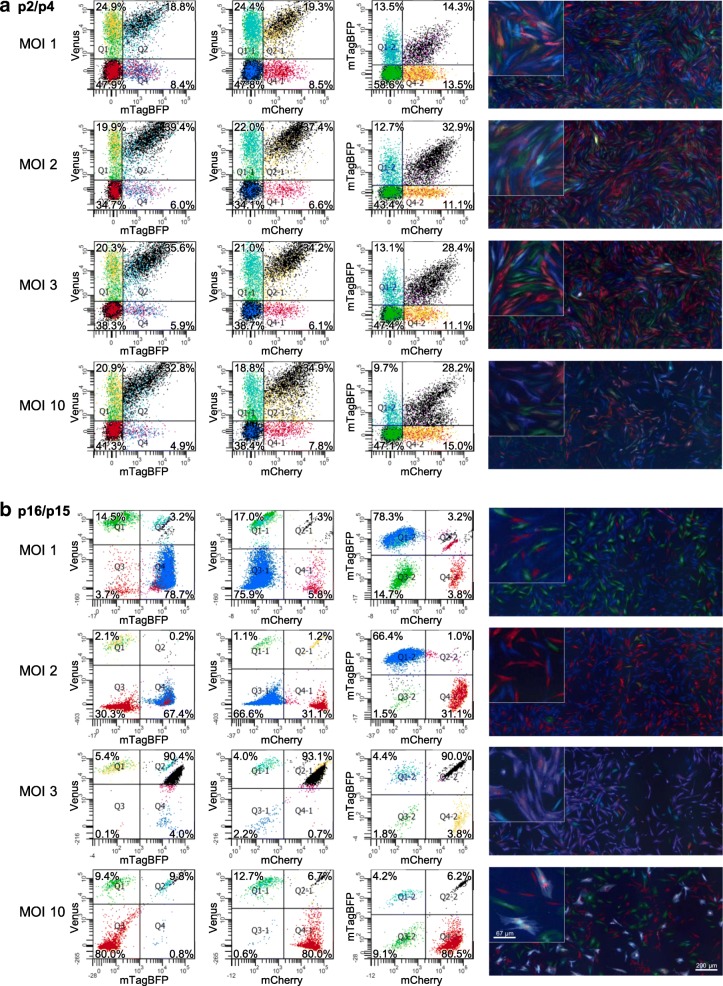
Flow cytometry and fluorescence microscopy images of primary human dermal fibroblasts kept in culture for 2 months after RGB marking at different MOI values. MOI 1, 2, 3 or 10 used to transduce primary human dermal fibroblasts. **a** Flow cytometry at passage 2 showing homogeneous polyclonal state of four independent cultures. Microscopic images taken at passage 4. **b** Flow cytometry at passage 16 showing oligoclonal state with few very dominant clones present at all four MOIs. Microscopic images taken at passage 15, dominant clones clearly identifiable by colour. Insets display higher magnification. Refer to Additional file 1: Figure S4 for a full data set from the four independent fibroblast cultures transduced at different MOI values. BFP blue fluorescent protein, MOI multiplicity of infection, p passage, Q quadrant

Similarly, human fibroblasts were also labelled with RGB marking and tracked over time. In this experiment, we already observed first dominant clones in the second flow cytometric analysis during the third passage after RGB marking (Fig. 4a). As observed for all other cell types, the polyclonal culture of human fibroblasts shifted to an oligoclonal state within weeks. In further passages, it was even possible to recognise the different clones in fluorescence microscopic images by their morphology in addition to the RGB marking, with very different cell sizes for the blue, red and green clones. The red clone consisted of small cells, the green clone displayed variable intermediate cell shapes, whereas the blue clone consisted of larger cells only (Fig. 4b and Additional file 1: Figure S4). This observation provided a very descriptive example of how powerful RGB marking can be to assess changes in clonality in human cell cultures, even after a period of time shorter than 1 month.

## Discussion

The importance of cell culture quality has been increasingly recognised in the stem cell field, ever since this area of research flourished during the past years [23]. The hopes raised by regenerative medicine with possible transplantation of laboratory-grown iPSCs into patients put considerable pressure on the field to produce large quantities of xeno-free genetically and epigenetically intact stem cells or stem-cell-derived cells. However, up to now surprisingly little emphasis has been placed on this field of research with a majority of iPSC studies simply assuming genetic and epigenetic stability and integrity over long-term cell culture.

Recently, however, more and more studies point out iPSC variability depending on multiple factors such as passage number, culture methods, reprogramming technique or origin of the cells, which would explain lab-to-lab and cell-line to cell-line variabilities [8, 24]. In the present study, we used RGB marking, a multicolour clonal tracking technique, to provide further evidence that potentially unfavourable clonal dynamics routinely happen during standard cell culture. The advantage of our tool is its simplicity and cost-effectiveness. It allows studying cellular dynamics with an easily interpretable output using fluorescence microscopy or flow cytometry.

Our results suggest that iPSC cultures start to become compromised after only 12–16 passages and sometimes even fewer. This effect will continue until passage 30 where one or two sub-clones from the initially marked population have overgrown 90% of the cell culture. Slower cell division seems to attenuate the effect—as seemed to be the case in C25-young cells in our experiments. In order to compensate for slower cell growth the splitting ratio was lowered, leading to more colours being kept from one passage to the other, in turn leading to a slower reduction of the clonality. The two other cell lines, however, were split at the same ratio and thus displayed similar speed in clonal competition. The same conclusion could be reached independently of the technique used to analyse the cells (fluorescence microscopy or flow cytometry), albeit with higher sensitivity in the flow cytometry analysis, which was able to detect reduced clone numbers earlier.

Three dominant clones arising from two of the iPS cell cultures were FACS sorted and karyotyped, and did not reveal any chromosomal abnormalities. These findings confirm results of other studies reporting more subtle genetic and epigenetic changes which cannot be detected by karyotyping [8]. Without the colours of RGB marking, all three iPSC cultures at the end of the experiment looked completely normal by light microscopy, without any noticeable evidence for the clonal drift.

These results could be reproduced in non-iPSC lines, confirming that clonal competition is probably taking place in each and any cell culture dish, as has been predicted by genetic barcoding of cell lines [25]. Our experiments showed that even in robust cell lines, often thought to be “monoclonal” per se, remarkable sub-clonal dynamics takes place, which would be invisible during routine cell culture. Within a few months of culture, sub-clones from only a few cells or even one single cell overgrew the whole culture (e.g. Fig. 4b, MOI 3), potentially leading to variable experimental results with these cells, explained by clonal instability. Interestingly, this observation could spark new experiments: Similar experiments with RGB-marked cells could be of special interest whenever a high selective pressure is applied to the cells, such as in cytotoxicity assays. Surviving clones could be FACS sorted based on their colour and characterised for survival factors or resistance mechanisms (e.g. against chemotherapeutic agents). The ability to identify and even sort viable cell clones is a great advantage over other methods often relying on cell lysis for clonal analyses. Even without strong selective pressure, our results might provide an interesting starting point to study tumour cell heterogeneity [26].

A possible solution to attenuate this effect whenever it is undesired, such as in iPSC culture, could be to culture cells in larger dishes in order to slow down clonal selection. Using a larger cell culture dish reduces the probability of discarding a sub-clone by chance, as a clone would be less likely to be only present in the discarded cells and thus be kept for further passage. However, this method would retain individual disadvantaged clones in culture for longer, but not attenuate growth advantages and disadvantages themselves. Furthermore, our experiments could not unambiguously prove that larger culture sizes led to a longer time before reaching oligoclonal states. Even the sole expansion of cells, without discarding any cells during replating, could lead to clonal imbalances because of different growth speed.

One caveat in our experiments remains that part or all of the observed sub-clonal dynamics could be due to lentiviral integration of the LeGO vectors. To test this hypothesis, one could use a culture with only half of the cells labelled with RGB marking and observe whether the transduced or the non-transduced cells are more likely to gain a competitive advantage over other cells. However, the probability of a retroviral vector integrating in a functional gene has been described as low as 1 × 10^− 8^–4 × 10^− 8^ per insertion [27]. Other methods to track clonality, such as lentiviral or CRISPR-based genetic barcoding, could also introduce a bias. Indeed, the same dynamics of sub-clonal overgrowth were observed in studies with genetic barcoding at an even lower lentiviral transduction rate [25]. Furthermore, three aspects in our experiments argue against insertional mutagenesis. First, using the physiological EF1α promoter and a self-inactivating lentiviral vector to label the iPS cells rendered insertional mutagenesis unlikely [28]. Second, the number of transduced cells was small, with 150,000 cells in the iPS cell experiments and as low as 50,000 cells in the 293 T/K562 experiments. This is not enough to expect several dominant clones to be generated by the aforementioned small number of insertion events. Finally, in the experiments with the 293 T and K562 cell lines, we used the same parental culture to start four different long-term cultures each (one T75 flask and three wells). If clonal dominance was a result of insertional mutagenesis, the same dominant clone should show up in all four cultures eventually, which we did not observe.

We conclude that sub-clonal dynamics routinely occur during culture of any cell type. The sub-clones could differ from others in terms of genetics, epigenetics and/or proteomics. We would therefore recommend culturing cells in larger flasks and to adhere to the principle of master and working cell banks to minimise the number of passaging cycles. It also might be necessary to discard cultures in which at any point during culture only a low amount of cells survived. Indeed, such a culture most likely lost most of its sub-clonal diversity, and even if it seems to recover well and can be further cultured, the quality of the culture might have been drastically changed. Moreover, it is recommended to keep the number of passages under 15 after the cell line has been established.

## Conclusions

Within weeks of routine iPS cell culture, one or a few of the initial clones overgrow the whole culture. Cells with relative growth advantage can be identified by RGB marking even when they are morphologically and karyotypically normal.

## Additional files

Additional file 1: **Figure S1.** Karyotyping. One clone from BJ cell line and two clones from C25-old cells sorted by flow cytometry, cultured for a few passages and karyotype analysed. Karyotyping indicated no chromosomal anomalies in the three sub-clones. **Figure S2.** Flow cytometry and fluorescence microscopy of RGB-marked cell line 293 T at different time points. Full data for cell line 293 T from Fig. 3. Left to right in each panel: green plotted against blue channel, green against red and blue against red. MOI 4 used to transduce 293 T cells. (**A**) Clonal dynamics in T75-flask. (**B–D**) Clonal dynamics in one individual well of a six-well cell culture plate over 25 weeks. (E) Stacked area plots displaying time course of proportion of each possible combination of colours (no colour, red only, green only, blue only, red and green, red and blue, blue and green, all three colours) to sum of all cells. Note similar dent in all plots at week 23 (right panel), probably due to technical reasons. Left panel shows plots with interpolated count at week 23. (**F**) Fluorescence microscopic images of four cultures in (A)–(D) at week 25. Images taken 3 days after replating. **Figure S3.** Flow cytometry and fluorescence microscopy of RGB-marked cell line K562 at different time points. Full data for cell line K562 from Fig. 3. Left to right in each panel: green plotted against blue channel, green against red and blue against red. MOI 16 used to transduce K562 cells. (**A**) Clonal dynamics in T75-flask. (**B–D**) Clonal dynamics in one individual well of a six-well cell culture plate over 25 weeks. (**E**) Stacked area plots displaying time course of proportion of each possible combination of colours (no colour, red only, green only, blue only, red and green, red and blue, blue and green, all three colours) to sum of all cells. Note similar dent in all plots at week 23 (right panel), probably due to technical reasons. Left panel shows plot with interpolated count at week 23. (**F**) Fluorescence microscopic images of the four cultures shown in (A)–(D) at week 25. Images taken 3 days after replating. **Figure S4.** Flow cytometry and fluorescence microscopic images of RGB-marked primary human dermal fibroblast cultures at different time points. Full data set for primary fibroblasts from Fig. 4. Left to right in each panel: green plotted against blue channel, green against red and blue against red. MOI 1, 2, 3 and 10 used for transduction and four cultures kept separately afterwards. (**A**) Clonal dynamics analysed by flow cytometry only (p2). (**B**) Clonal dynamics analysed by flow cytometry and imaged by fluorescence microscopy (further passages). Insets display higher magnification (PDF 24080 kb)

Additional file 2: **Video S1.** Video-optical recording of RGB-marked EHT. The EHT displayed unaltered contractility with similar force and frequency of contraction as unmarked controls (MP4 7660 kb)

## Abbreviations

ATCC: American Type Culture Collection
bFGF: Basic fibroblast growth factor
BFP: Blue fluorescent protein
BMP4: Bone morphogenic protein 4
CML: Chronic myeloic leukaemia
DMEM: Dulbecco’s modified Eagle’s medium
EB: Embryoid body
EDTA: Ethylenediaminetetraacetic acid
EF1α: Eukaryotic elongation factor 1α
EHT: Engineered heart tissue
ES: Embryonic stem
FTDA: FGF, TGF-β1, dorsomorphin, activin A
HDFa: Human dermal fibroblast
hiPS: Human induced pluripotent stem
LeGO: Lentiviral Gene Ontology
MOI: Multiplicity of infection
RGB: Red, green, blue
RPMI: Roswell Park Memorial Institute medium
SFFV: Spleen focus-forming virus
TGF-β1: Transforming growth factor β1
VSV-G: Vesicular stomatitis-virus glyco-protein
