# Supplementary material for: Clonal dynamics studied in cultured induced pluripotent stem cells reveal major growth imbalances within a few weeks
Source: Stem Cell Res Ther. 2018 Jun 18;9:165. doi: 10.1186/s13287-018-0893-2 (PMC6006556; doi:10.1186/s13287-018-0893-2)
Supplement: Supplementary file 1 — Figure S1. Karyotyping. One clone from BJ cell line and two clones from C25-old cells sorted by flow cytometry, cultured for a few passages and karyotype analysed. Karyotyping indicated no chromosomal anomalies in the three sub-clones. Figure S2. Flow cytometry and fluorescence microscopy of RGB-marked cell line 293 T at different time points. Full data for cell line 293 T from Fig. 3. Left to right in each panel: green plotted against blue channel, green against red and blue against red. MOI 4 used to transduce 293 T cells. (A) Clonal dynamics in T75-flask. (B–D) Clonal dynamics in one individual well of a six-well cell culture plate over 25 weeks. (E) Stacked area plots displaying time course of proportion of each possible combination of colours (no colour, red only, green only, blue only, red and green, red and blue, blue and green, all three colours) to sum of all cells. Note similar dent in all plots at week 23 (right panel), probably due to technical reasons. Left panel shows plots with interpolated count at week 23. (F) Fluorescence microscopic images of four cultures in (A)–(D) at week 25. Images taken 3 days after replating. Figure S3. Flow cytometry and fluorescence microscopy of RGB-marked cell line K562 at different time points. Full data for cell line K562 from Fig. 3. Left to right in each panel: green plotted against blue channel, green against red and blue against red. MOI 16 used to transduce K562 cells. (A) Clonal dynamics in T75-flask. (B–D) Clonal dynamics in one individual well of a six-well cell culture plate over 25 weeks. (E) Stacked area plots displaying time course of proportion of each possible combination of colours (no colour, red only, green only, blue only, red and green, red and blue, blue and green, all three colours) to sum of all cells. Note similar dent in all plots at week 23 (right panel), probably due to technical reasons. Left panel shows plot with interpolated count at week 23. (F) Fluorescence microscopic images of the four [file 13287_2018_893_MOESM1_ESM.pdf]

Fig. S1

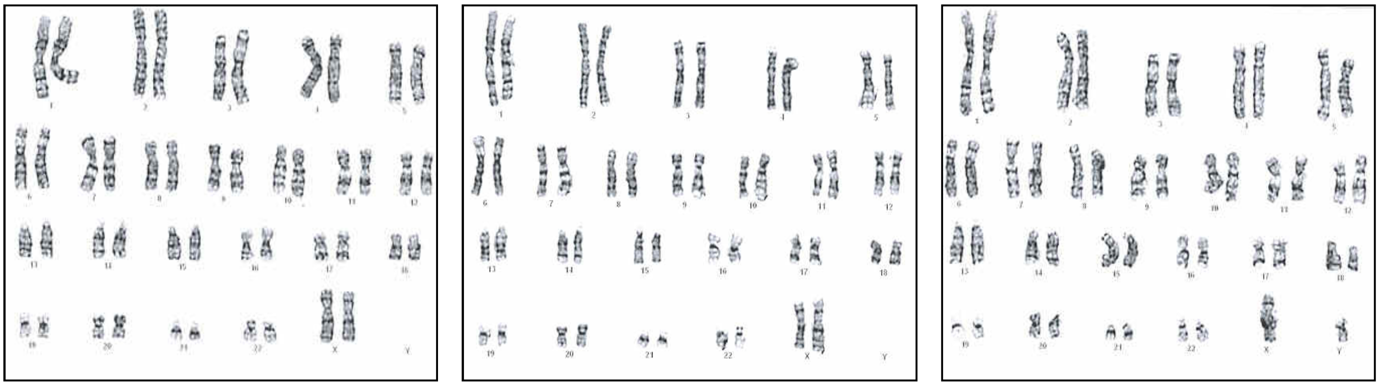

C25-old #1 **C25-old #2** **BJ #1**

Fig. S2 A

293T cells
T75 flask

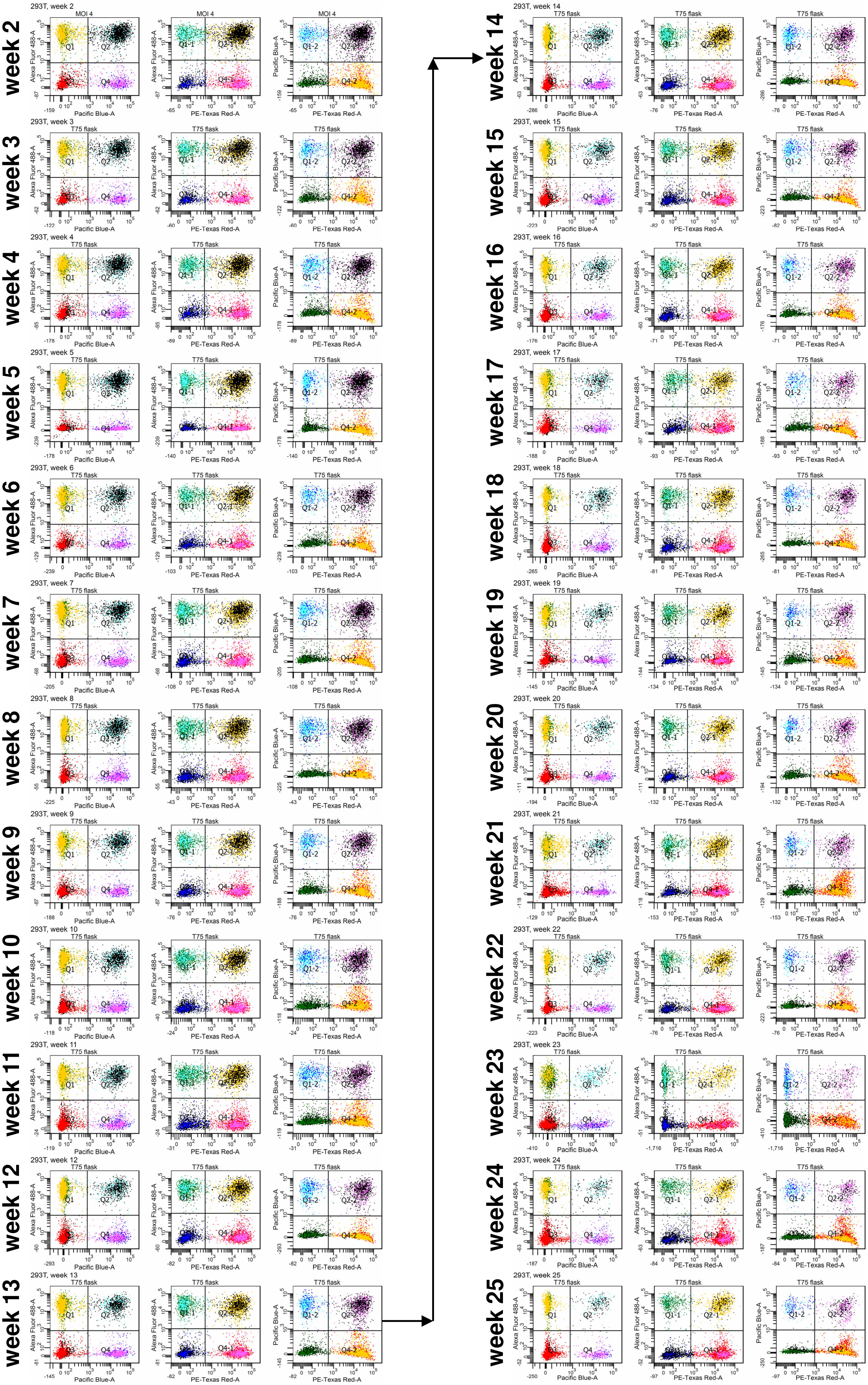

Fig. S2 B

293T cells
6-well A

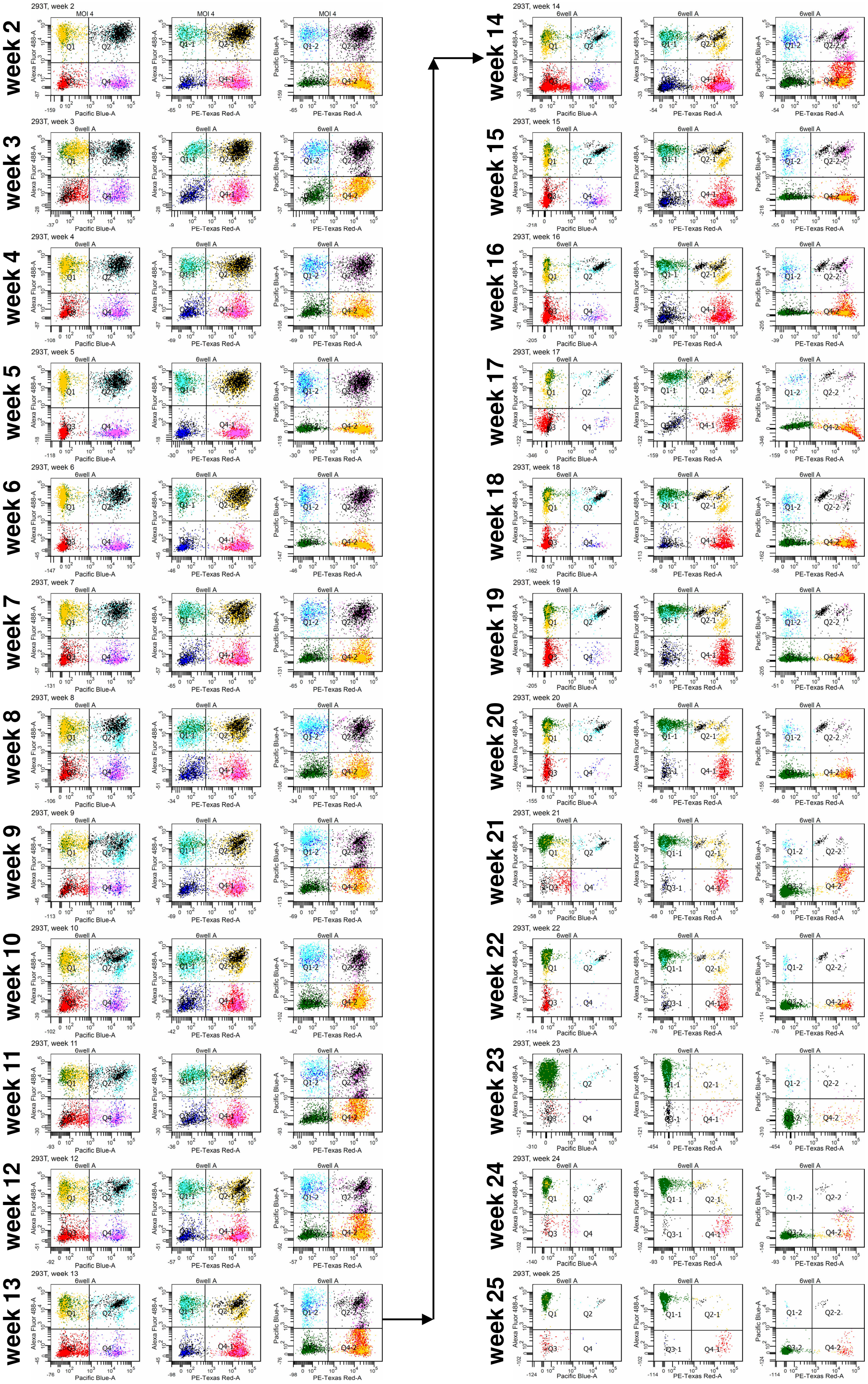

Fig. S2 C

293T cells 6-well B

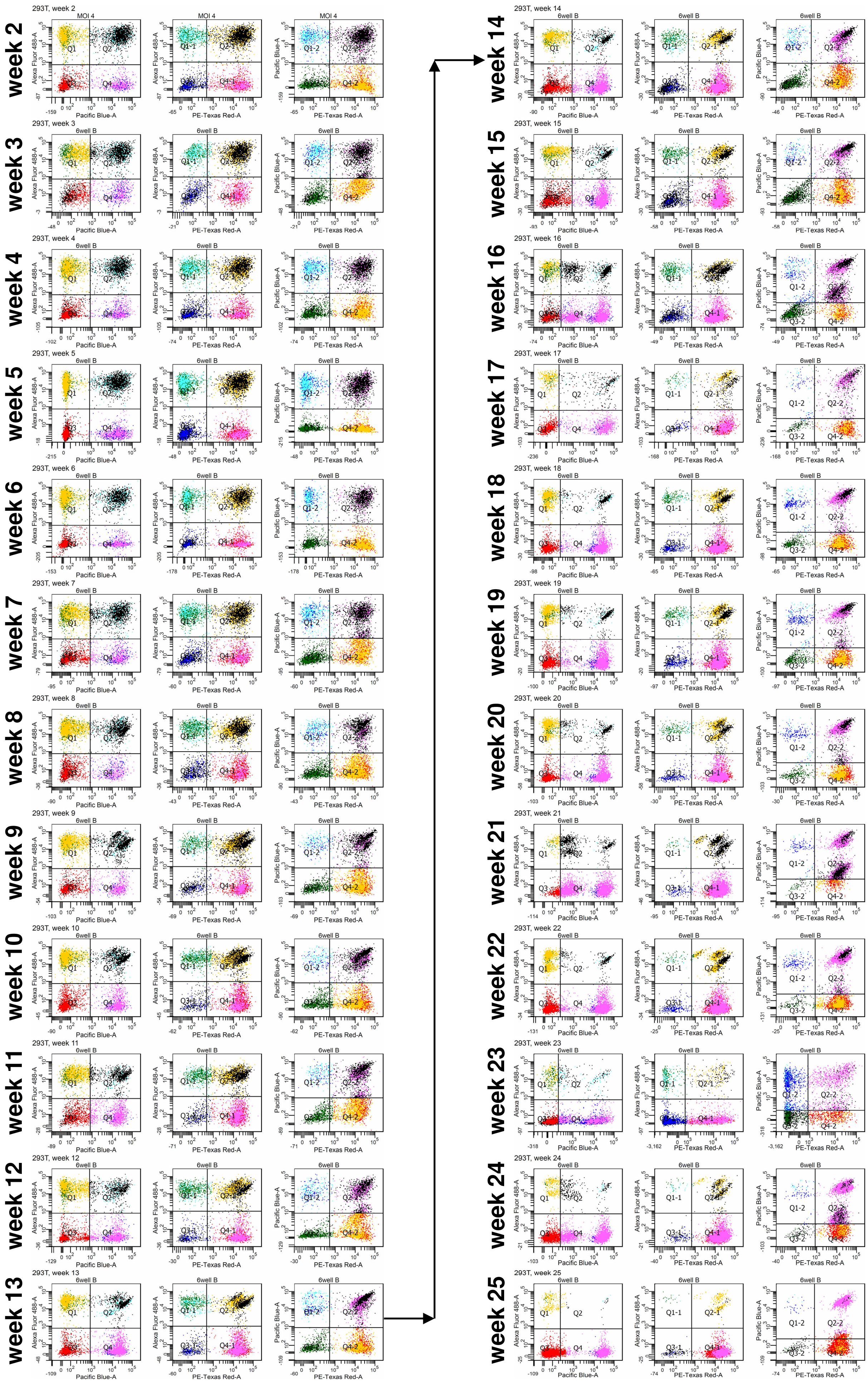

Fig. S2 D

293T cells
6-well C

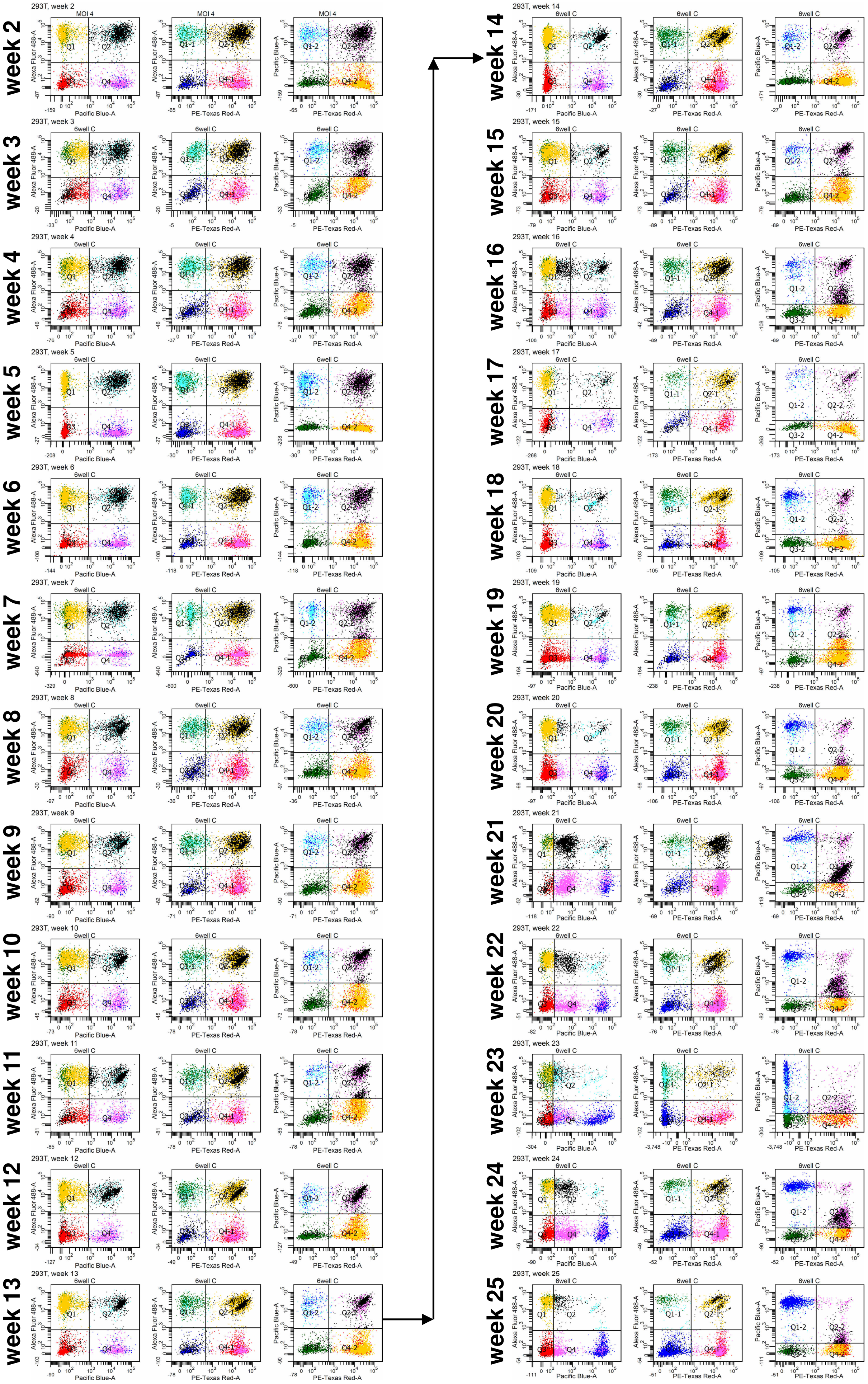

Fig. S2 E Stacked area plots summing up 293T FACS data of Fig. S2 A-D

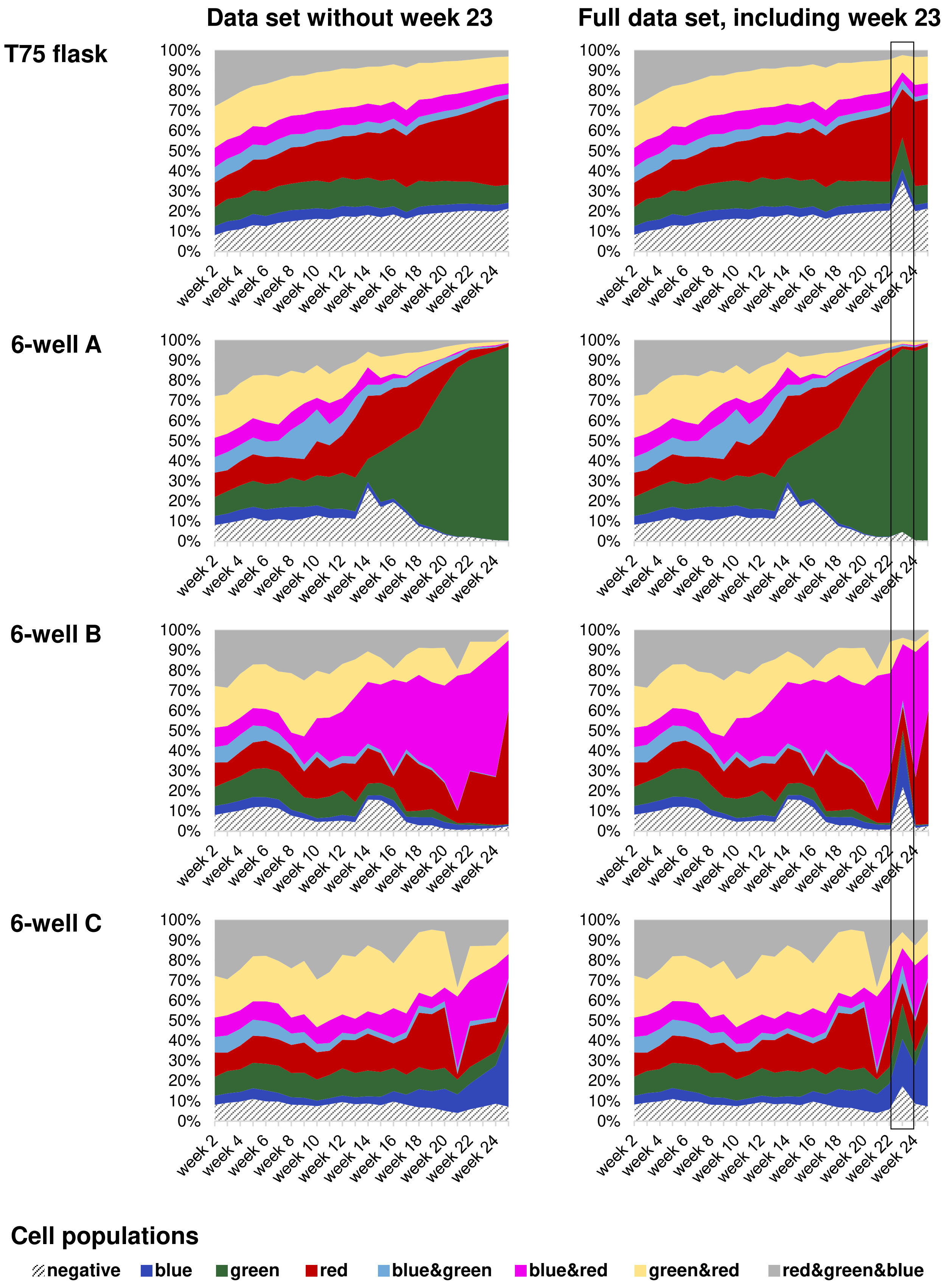

Fig. S2 F   293T cells at the end of the experiment (week 25)

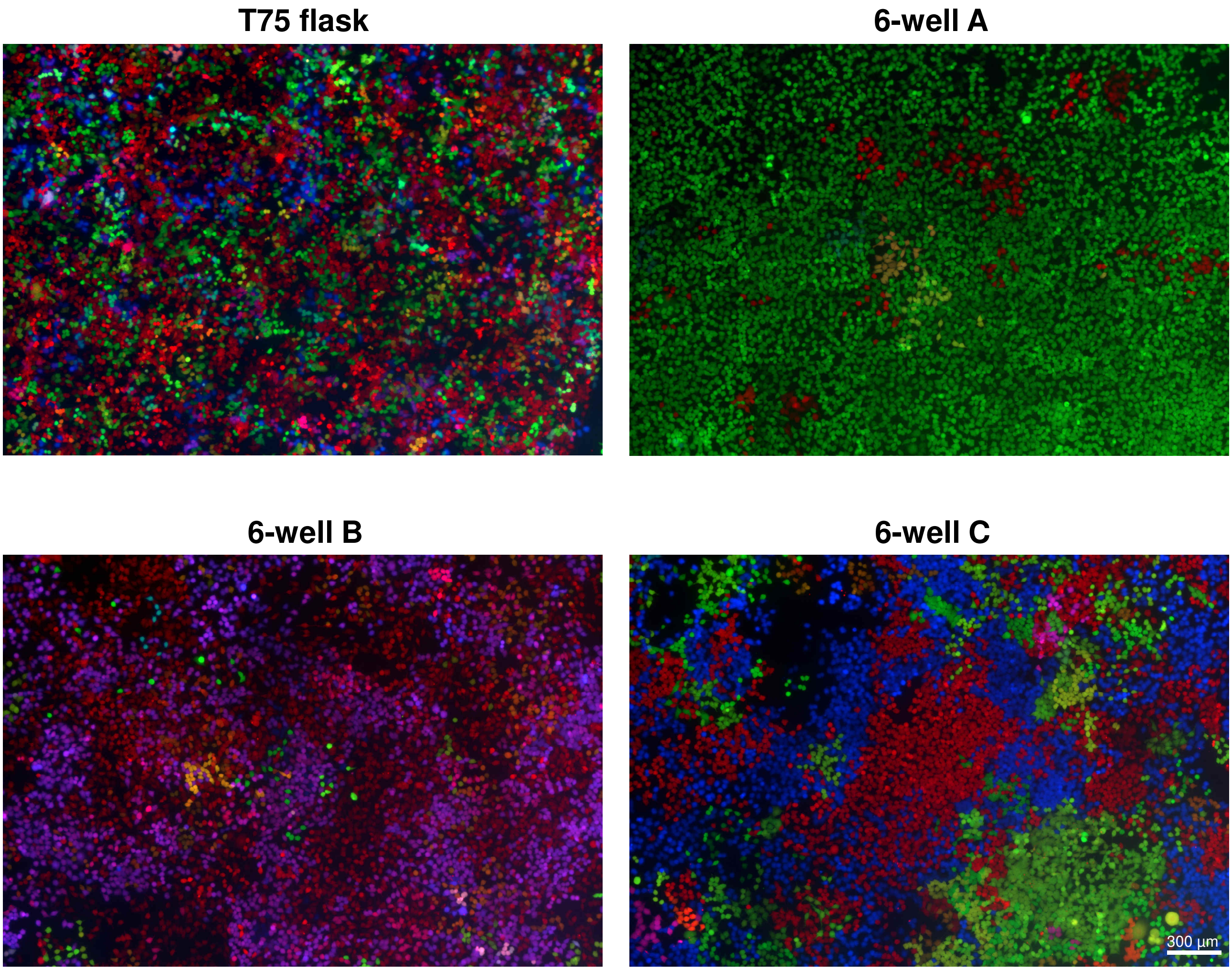

Fig. S3 A

K562 cells T75 flask

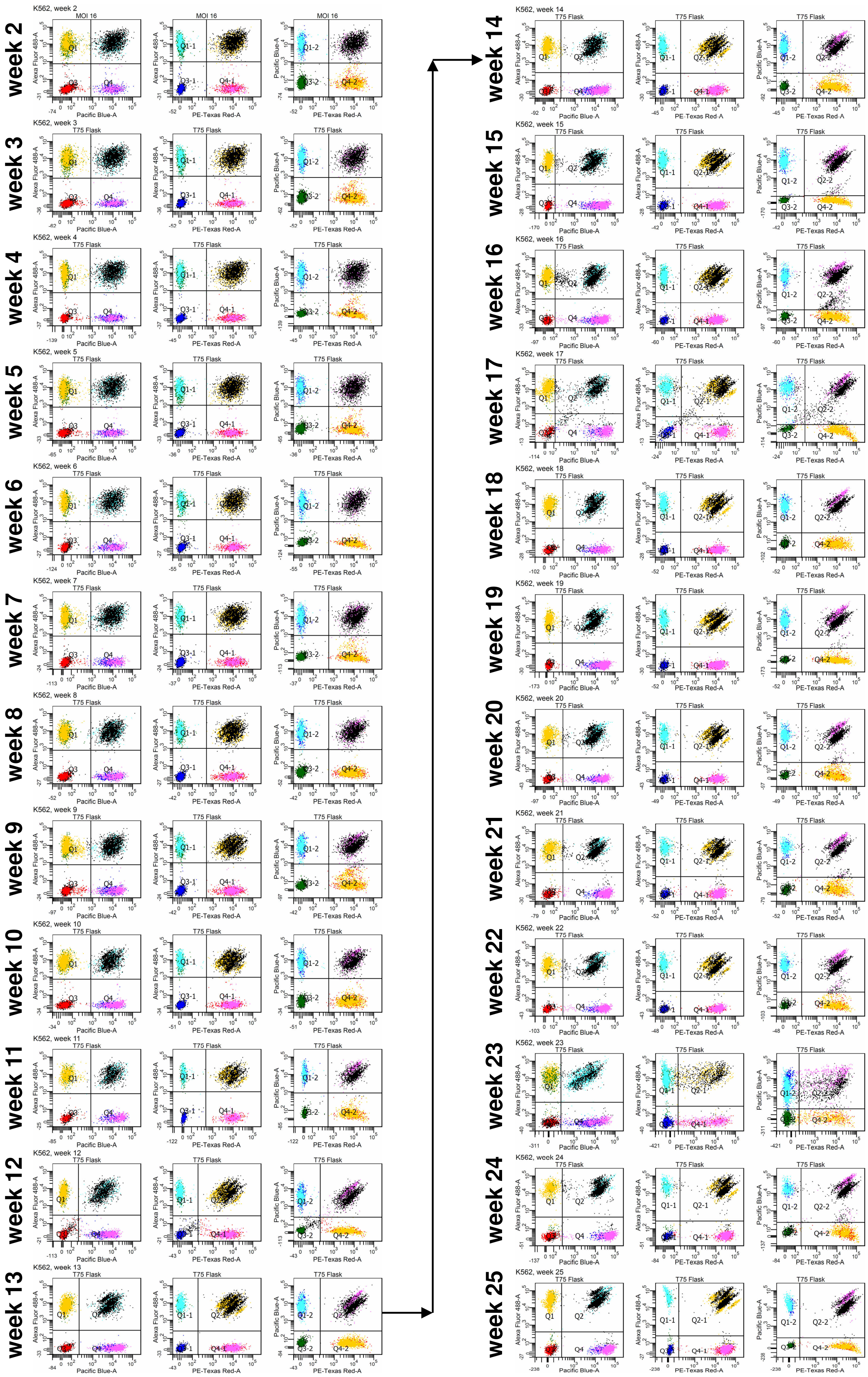

Fig. S3 B

K562 cells
6-well A

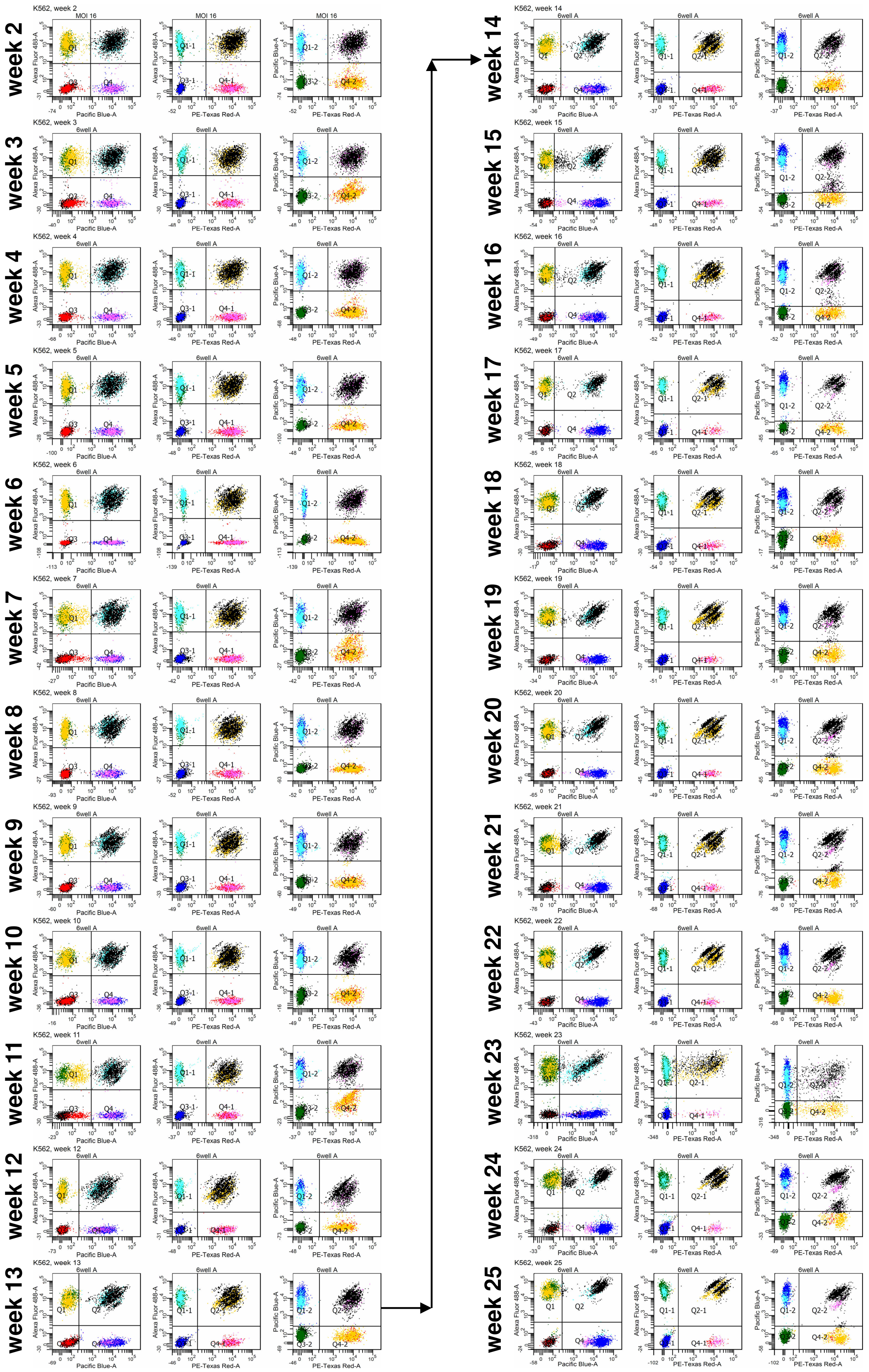

Fig. S3 C

K562 cells 6-well B

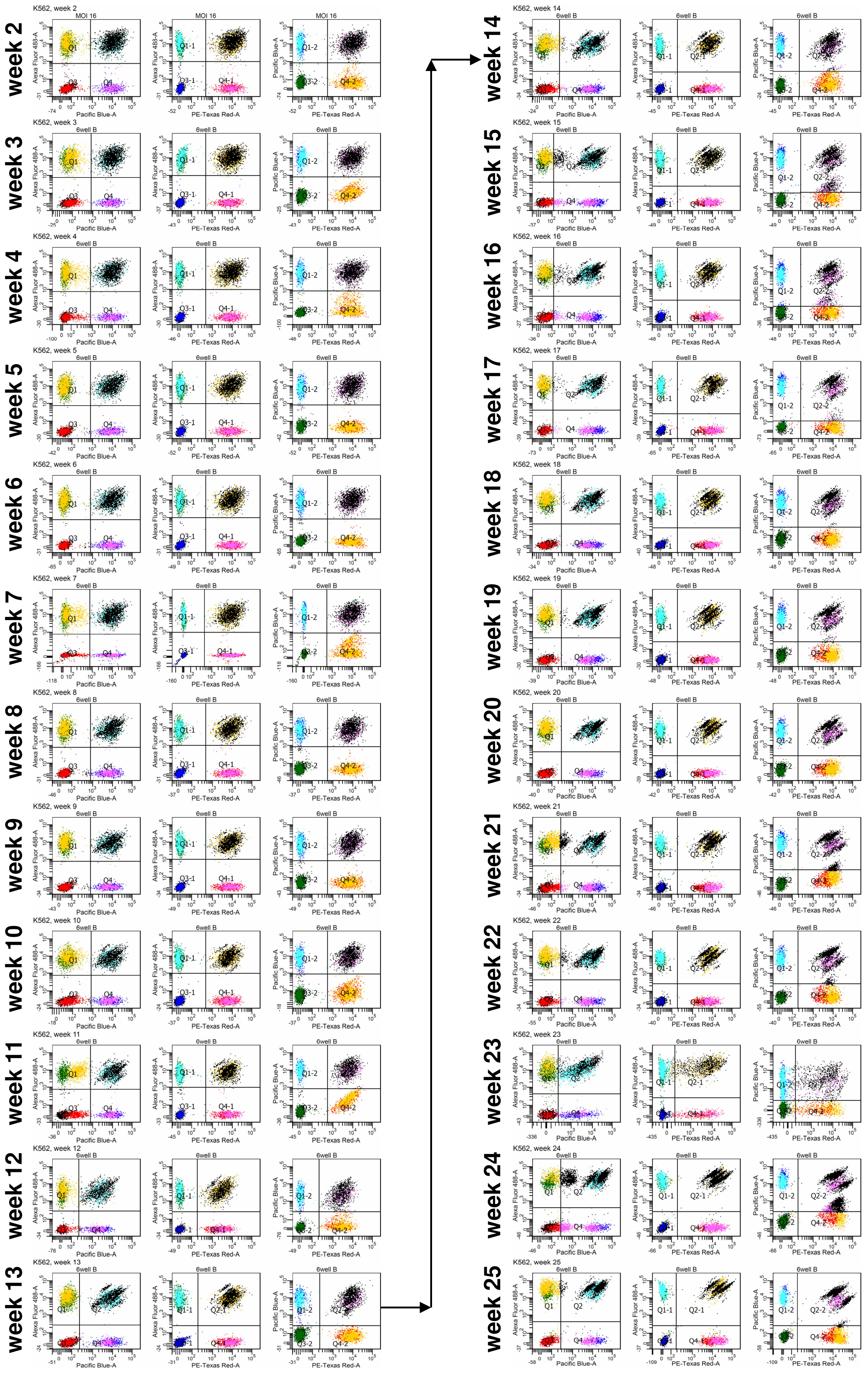

Fig. S3 D

K562 cells 6-well C

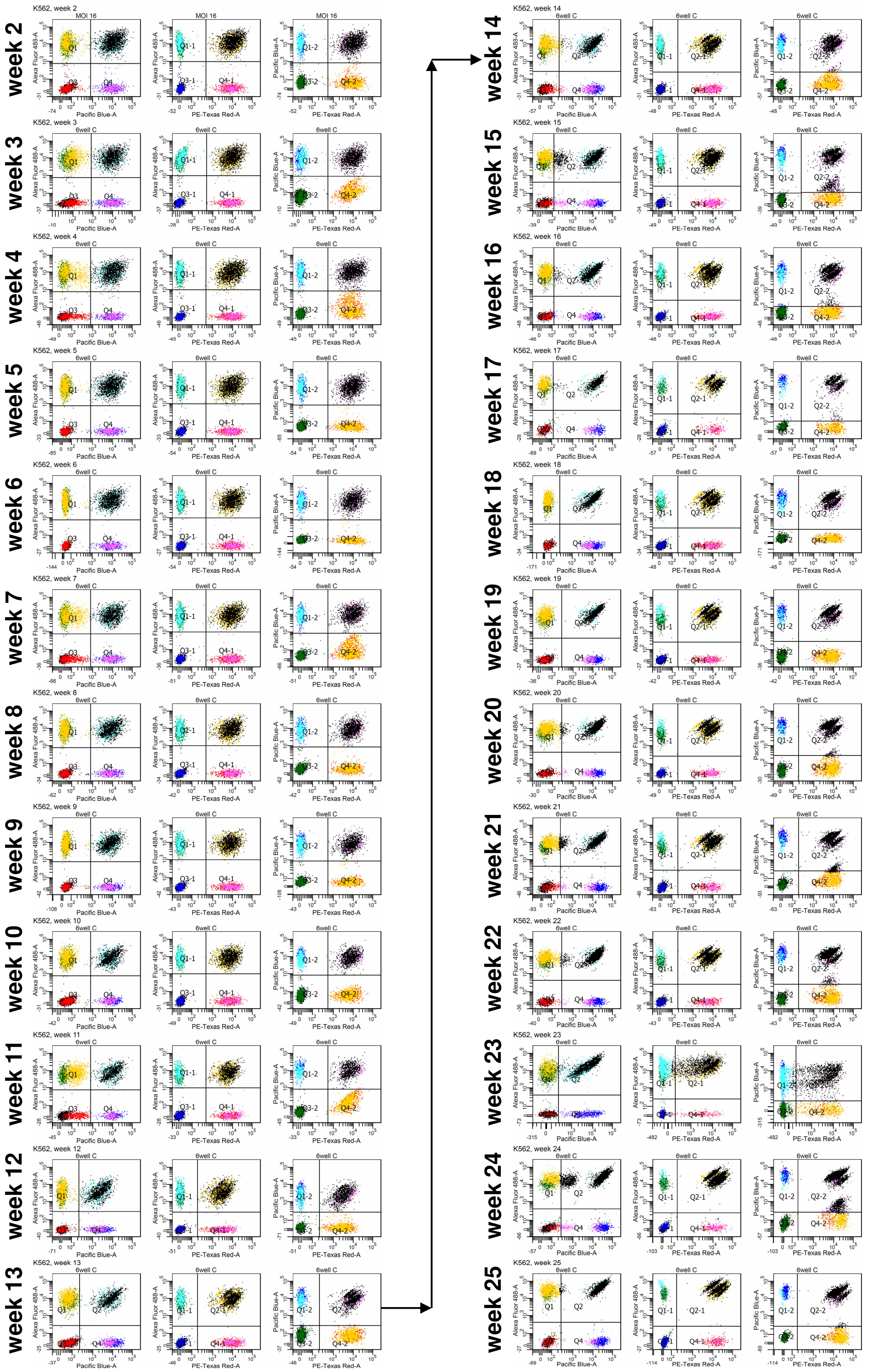

Fig. S3 E   Stacked area plots summing up K562 FACS data of Fig. S3 A-D

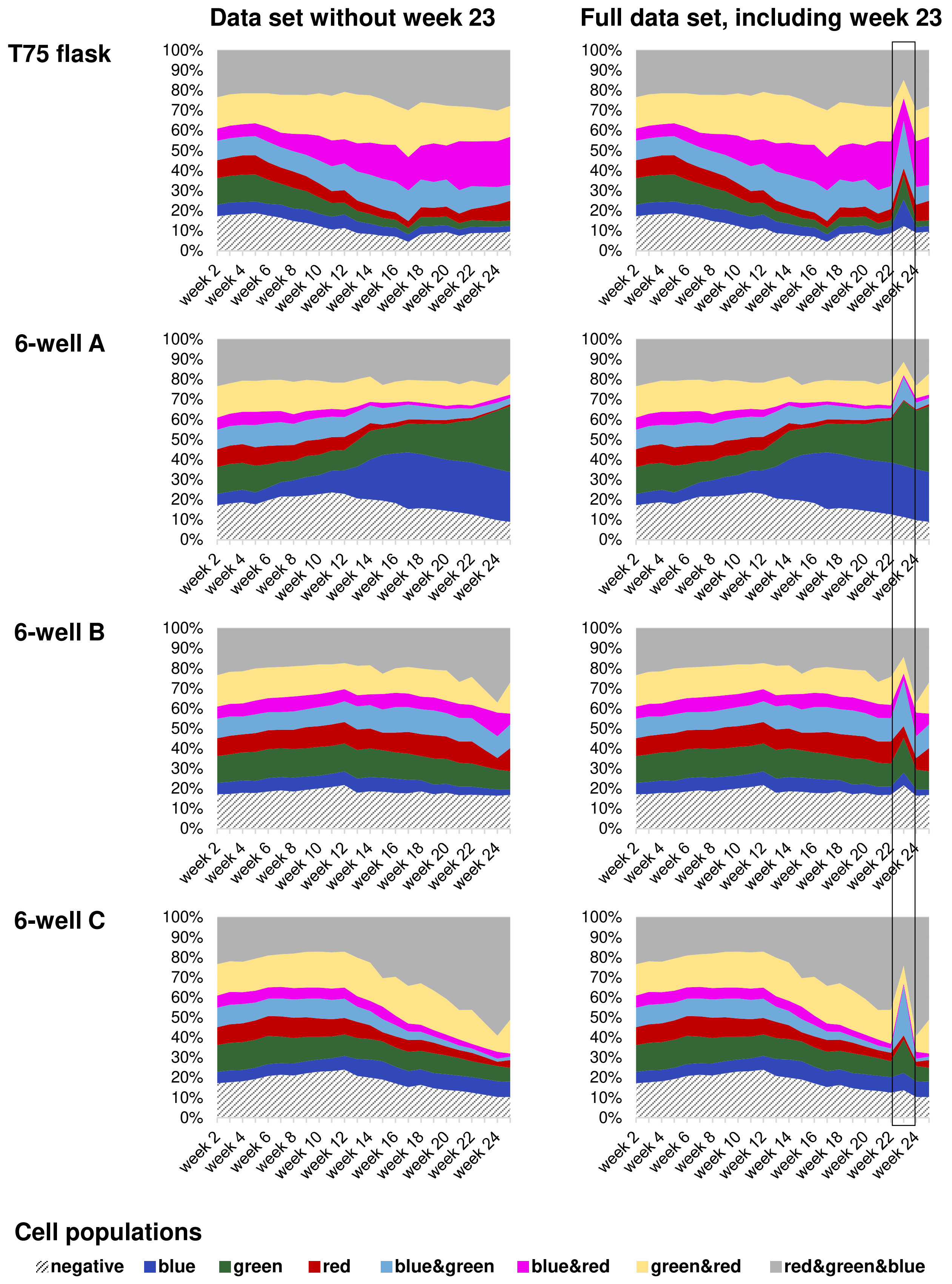

Fig. S3 F   K562 cells at the end of the experiment (week 25)

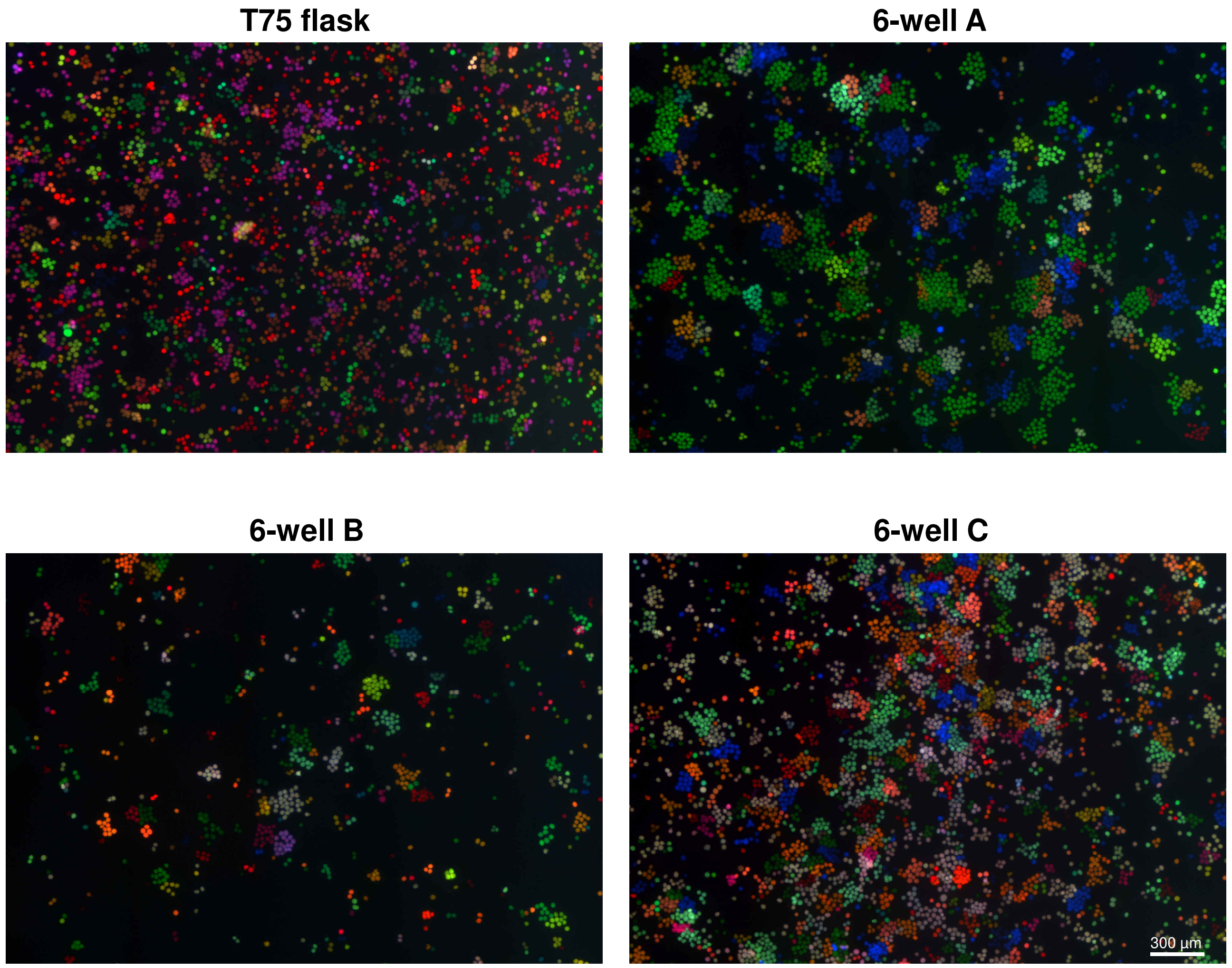

Fig. S4

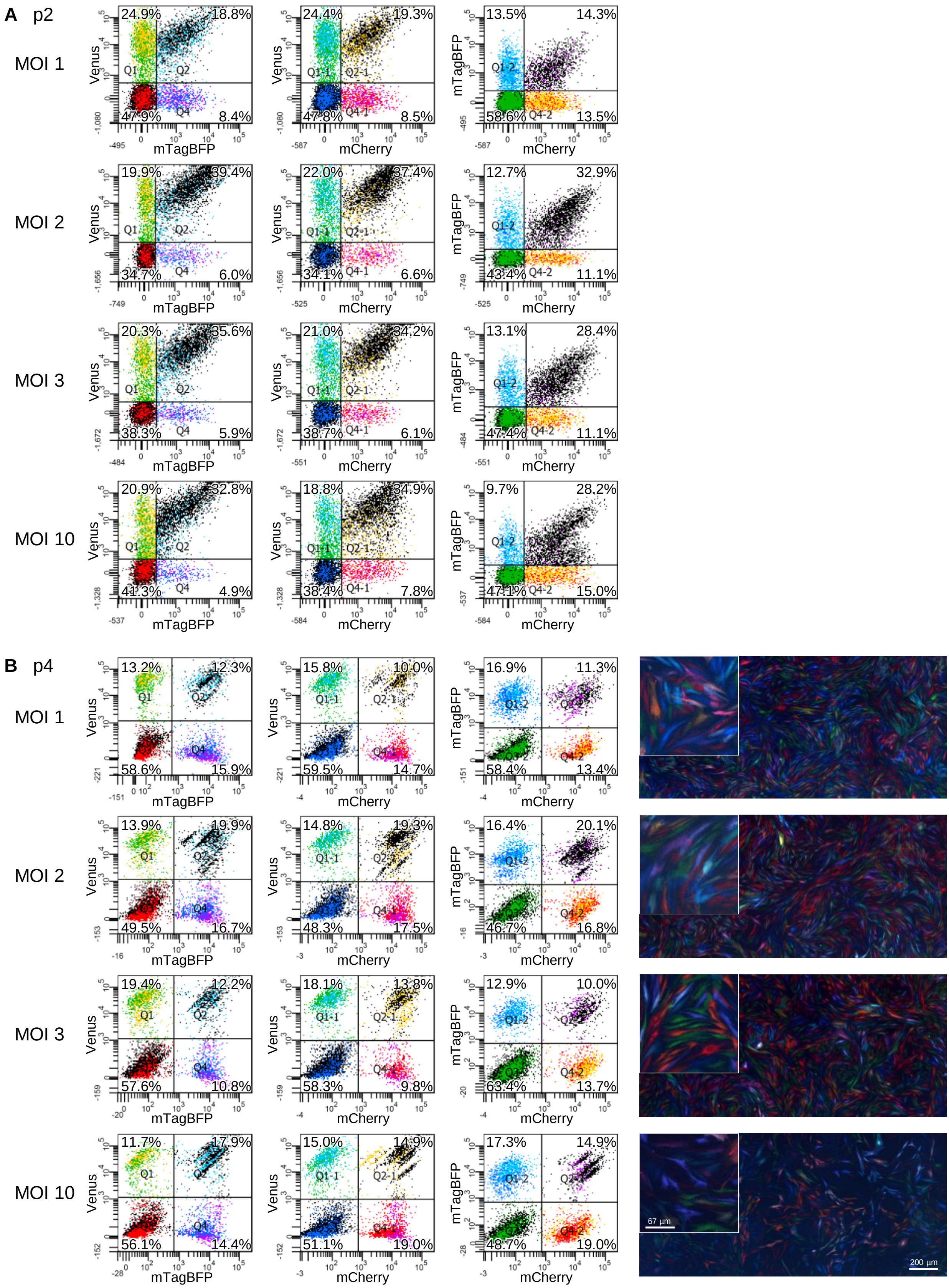
A
p2
MOI 1
MOI 2
MOI 3
MOI 10
B
p4
Venus
mTagBFP
mCherry
67 µm
200 µm

Fig. S4

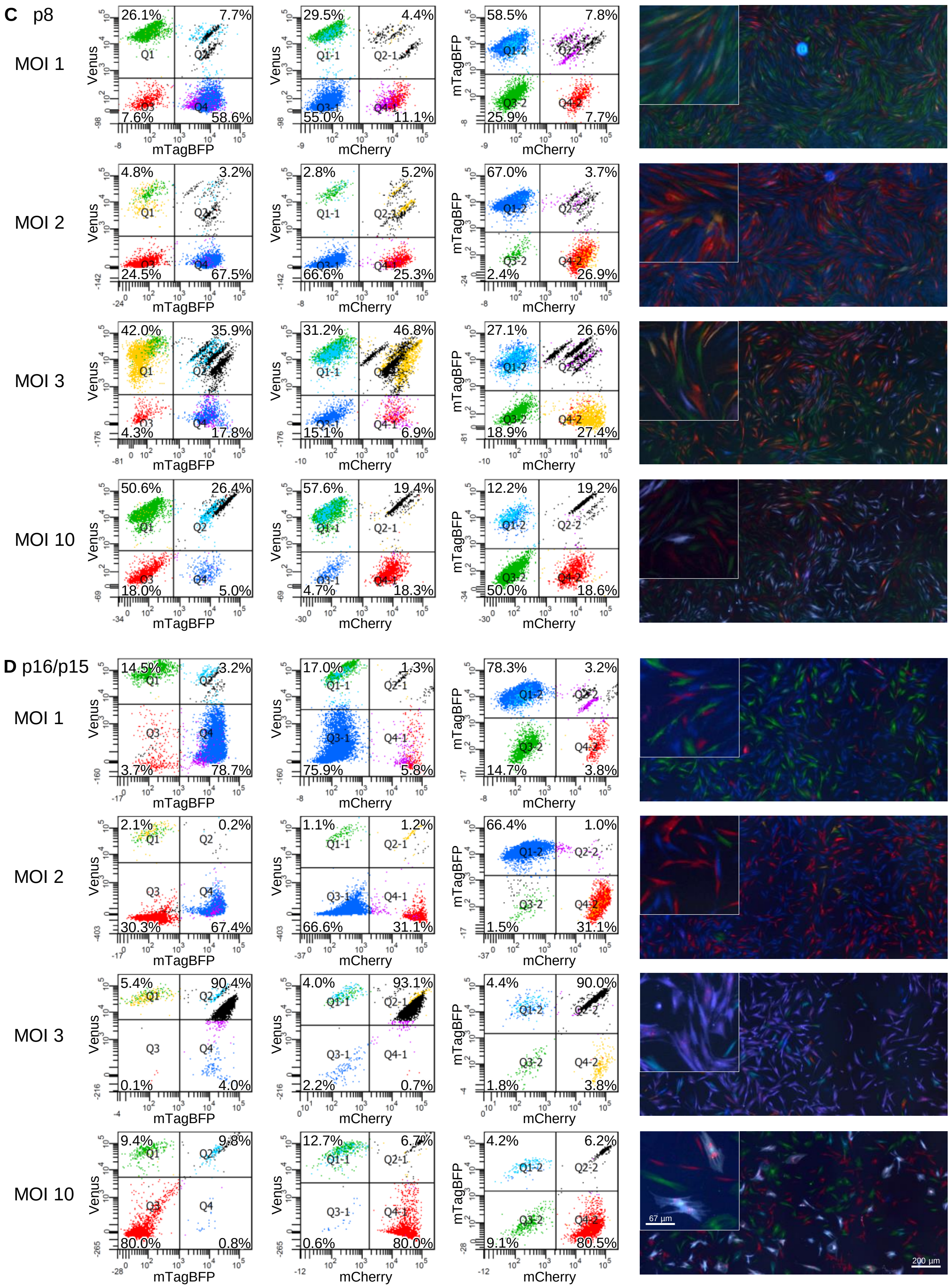
C p8
D p16/p15
MOI 1
MOI 2
MOI 3
MOI 10
Venus
mTagBFP
mCherry
67 µm
200 µm
